# Psychological stress has a higher rate of developing addictive behaviors compared to physical stress in rat offspring

**DOI:** 10.17179/excli2016-685

**Published:** 2017-06-19

**Authors:** Masoud Nazeri, Arezoo Ebrahimi, Iraj Aghaei, Samaneh Ghotbi Ravandi, Mohammad Shabani

**Affiliations:** 1 Department of Oral Medicine and Chronic Headache and Facial Pain Clinic, School of Dentistry, Kerman, IranUniversity of Medical Sciences, Kerman, Iran; 2 Neuroscience Research Center, Neuropharmacology Institute, Kerman University of Medical Sciences, Kerman, Iran; 3 Social Determinants of Health Research Center, Gilan University of Medical Sciences, Rasht, Iran

**Keywords:** prenatal stress, physical stress, psychological stress, morphine preference, anxiety-like behavior

## Abstract

Prenatal stress could have great influence on development of offspring and might alter cognitive function and other physiological processes of children. The current study was conducted to study the effect of physical or psychological prenatal stress on addictive and anxiety-like behavior of male and female offspring during their adolescence period (postnatal day (PND) 40). Adult female rats were exposed to physical (swimming) or psychological (observing another female rat swimming) stress from day six of gestation for 10 days. Male and female offspring were assayed for anxiety-like behavior, motor and balance function and morphine conditioned place preference using the open field, elevated plus maze (EPM), rotarod and wire grip assay and conditioned place preference. Offspring in both physical and psychological prenatal stress groups demonstrated significant increase in anxiety-like behavior in EPM paradigm, but no alterations were observed in motor and balance function of animals. Offspring in the psychological prenatal stress group had an increased preference for morphine in comparison to control and physical prenatal stress groups. Results of the current study demonstrated that animals exposed to psychological stress during fetal development are at a higher risk of developing addictive behaviors. Further research might elucidate the exact mechanisms involved to provide better preventive and therapeutic interventions.

## Abbreviations

Elevated Plus Maze (EPM); Open Field (OFT); Conditioned Place Preference (CPP); Total Distance Moved (TDM)

## Introduction

Stress has long been considered as a contributing factor to the development of addictive behavior and relapse in humans and other animals (al'Absi, 2006[4]; Sinha, 2007[38]). It is proposed that stress during pregnancy would lead to alterations of the hypothalamic-pituitary-adrenal axis and makes offspring vulnerable to addiction (Henry et al., 1995[12]; Koehl et al., 2000[14]; Yang et al., 2006[45]; Kippin et al., 2008[13]; Thomas et al., 2009[41]).

There are a vast body of research supporting the role of stress in vulnerability to addiction and drug abuse (Koob, 1999[17], 2008[16]; Sinha, 2008[37]). Though there is a well-accepted role for stress in development of addiction, the exact neurobiological mechanisms are still unclear (Piazza and Le Moal, 1998[28]; Goeders, 2002[9], 2003[10]). The same is true for the role of prenatal stress in development of addiction in offspring (Rokyta et al., 2008[32]). 

Previous studies have demonstrated that various stressors differentially affect the neural circuitry of the brain, each stressor has its own specific effect on the brain (Muhammad and Kolb, 2011[24]; Nazeri et al., 2015[26]; Saboory et al., 2015[33]), and different neurobiological mechanisms correspond for such alterations (Swendsen and Le Moal, 2011[40]). Whether psychological or physical prenatal stress have the same effect on addictive behaviors or not is a matter of debate and human studies have demonstrated that the type of stressor is an important factor that affects the neural circuitry responsible for addiction (Sinha et al., 2000[39]; Gerra et al., 2001[7][8]). There is also a strong evidence for the role of sex hormones in developing addictive behaviors (Nelson-Zlupko et al., 1995[27]; Tuchman, 2010[42]) and response to stressors (Cooper et al., 1997[6]; Matud, 2004[23]).

According to previous studies and the lack of enough animal research regarding the effect of stressor type during pregnancy on addictive behavior, this study was designed to evaluate the effect of these two factors on addictive behavior in an animal model of addiction to morphine. A previously established animal model for physical or psychological stress was used in the current study (Nazeri et al., 2015[26]) to evaluate the effects of prenatal physical or psychological stress on offspring preference to morphine. Results of the current study might provide a platform for further research on the effects of prenatal stress on addictive behaviors of offspring.

## Methods and Materials

Adult female Wistar rats (weighing 220-250 g) were used for the current study. Animals were housed in cages of three with access to food and water ad-libitum. Animals were kept in standard conditions (room temperature 24 ± 2 °C, Light/dark cycle: 12/12h). All the procedures were approved by ethics committee and maximum effort was made to minimize the harm to the animals (Ethics code: KNRC/93-7).

### Application of prenatal stress

Female rats were introduced to a sexually active adult male rat in their first day of estrous cycle. To verify mating, samples were made from the vaginal secretion to ensure mating. The stress procedure commenced at sixth day of gestation and continued for the next 10 days using the following protocol.

**Figure 1:**
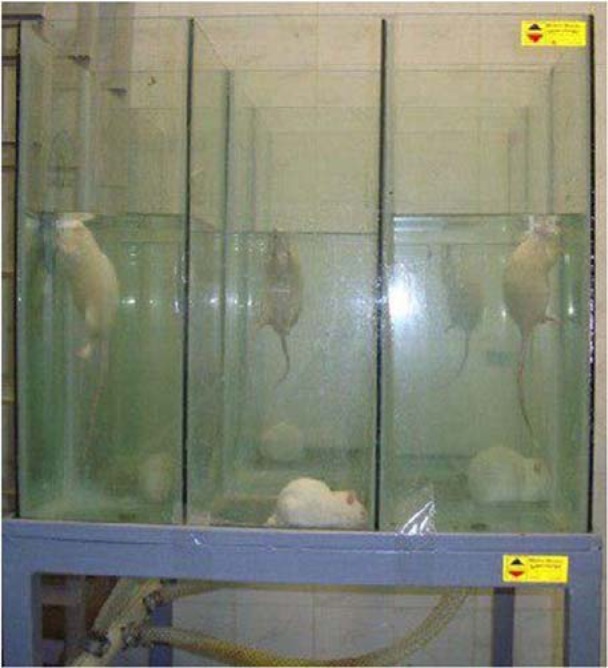
Communication stress box| A 3*3 transparent glass house aquarium (25 * 25 * 60 cm) containing five cells of physically stressed rats which is connected to an electric pump and four cells containing psychologically stressed rats. Each psychologically stressed rat has been surrounded by 3 physically stressed rats in order to induce emotional stress.

A previously established method was used to confront the pregnant animals with physical or psychological stress (Nazeri et al., 2015[26]; Aghaei et al., 2017[2]). Stressed rats were then exposed to two different stressors. A three*three compartment glass house aquarium (25 * 25 * 60 cm^3^ in volume) was used to stress the animals physically or psychologically so that each psychologically stressed rat was surrounded by three physically stressed rats (see Figure 1(Fig. 1)). A box of 9 cells that have been separated from each other by a Plexiglas was designed. This glass is transparent, which allows rats of different cells to see each other. Each of these 5 cells (containing physically stressed rats) was connected to an electric pump. The pump filled the cells with water (2±20 °C), which forced the rats to swim in the chamber. Animals receiving physical stress were placed in the houses of the aquarium that were filled with water and animals were forced to swim in the water for five minutes. Then the house drained and the animals could rest for ten minutes. Subsequently, the house was filled with water again. This procedure lasted for one hour. Psychological stress group rats were placed in the houses of aquarium that was not connected to the pump and just observed the other animals receiving physical stress of swimming. Swim stress is a known stressor which has already been shown to elicit stress response (Quintero et al., 2000[30]; Nazeri et al., 2014[25]). Animals were then dried with a clean dry towel and moved to their cages. As mentioned, this procedure lasted for the next 10 gestation days. There was also a control group in which, female rats were brought to the laboratory and remained in there for the same duration that stress groups rats were kept (Nazeri et al., 2015[26]).

There were 10 female rats in each study group (physical or psychological stress). Pups were delivered and the number of offspring, their mortality rate and birth weight was recorded for each animal. Two male and two female offspring were selected from each litter (n=20 for each group) and then were caged in groups of four. The behavioral tests were performed on postnatal day 40 (PND 40) (Nazeri et al., 2015[26]).

Offspring were brought to the laboratory one week before the initiation of behavioral assays to accommodate to the environment. On PND40, the following behavioral assays with a two hour interval between each test was performed on each rat: open field, elevated plus maze, rotarod and wire grip. 

### Open Field Test (OFT)

This test was recruited to evaluate the possible effect of prenatal physical or psychological stress on locomotion and anxiety-like behaviors. The apparatus consisted of an arena made of opaque Plexiglass (90 * 90 * 45 [H] cm). Rats were placed in the middle of OFT arena and their behavior was recorded by a video tracking system (Ethovision, Noldus technology, Netherlands). Total Distance Moved (TDM), velocity, total time spent in center and periphery was recorded for each rat. At the end of each experiment, the animals were removed from the arena and the OFT was cleaned with a cotton cloth and ethanol 10 % and the next animal was placed in OFT (Aghaei et al., 2014[3]).

### Elevated Plus Maze (EPM)

This assay was used to evaluate the anxiety-like behavior of the animals. The EPM was made of wood and had two open arms and two close arms (50 * 50 cm). Animals were placed in the middle of the maze and total distance moved (TDM), velocity, number of entrance into the open and close arms and time spent in each arm was recorded in a five minute duration using a video camera installed above the EPM (Nazeri et al., 2015[26]). 

### Rotarod

This test was used to assay the possible effects of stress on motor function and balance in the animals. An accelerating rotarod apparatus (Hugo Sachs Electronik, Germany) was used in the current assay. Each trial started from a speed of 10 rounds per minute (rpm) and reached a maximum speed of 60 rpm. Three trials were performed for each rat (maximum duration= 300 sec) with a 30 minute interval between each trial. The duration that each rat could stay on the rotarod and do not fall was recorded as a measure of balance and motor coordination (Razavinasab et al., 2013[31]).

### Wire grip

This assay was used to evaluate the muscle strength of the animals. Rats in a vertical posture were suspended on a horizontal steel wire hanging on both forepaws (80 cm long, diameter 7 mm). The rat was released whenever it grasped the wire. The latency to fall was recorded for each animal using a stop watch. Each rat underwent three trials with a 30 minute inter-trial interval (Shabani et al., 2012[36]).

### Conditioned Place Preference (CPP)

The day after behavioral assay, CPP was performed to evaluate the animals' preference for morphine use. The unbiased CPP animal model of morphine preference was recruited in the current study. The CPP chamber is made of three distinct compartments and each part's wall is covered by vertical lines (2 cm width; vertical compartment), horizontal lines (2 cm width, horizontal compartment) and white (neutral compartment). The compartments are separated by guillotine doors. Animals are placed in the neutral part in day one (pre-conditioning) and guillotine doors are removed to allow the animal to explore the compartments. Time spent in each part was recorded during a 20 minute period using a video camera installed above the CPP chamber and if the animals spent a relatively equal time in three compartments (not more than 60 % of time spent in one compartment), they were included in the study.

For the other next four days, subcutaneous injection of either saline or morphine (10 mg/kg dissolved in normal saline) was made for each animal so that morphine injection was associated with placement of the animals in the vertical compartment. After injection of morphine or saline, animals were placed in the respective compartment for 50 minutes (Afarinesh et al., 2008[1]; Haghparast et al., 2013[11]). 

On the sixth day, animals were again put in the neutral compartment and guillotine doors were removed to let the animal freely move through the compartments (post-conditioning). Time spent in each compartment was recorded. CPP score was calculated by using the following formula:

CPP score= time spent in vertical compartment in post-conditioning day - time spent in vertical compartment in pre-condition day. 

### Statistical analysis

Data were collected and analyzed by SPSS V.16 (IBM, USA). Two ways ANOVA followed by Tukey's post-hoc analysis was used to compare the mean level of different parameters amongst groups with considering of sex effects. P < 0.05 was considered statistically significant. 

## Results

### Effect of prenatal stress on locomotion and anxiety-like behaviors

#### Open field

**Figure 2:**
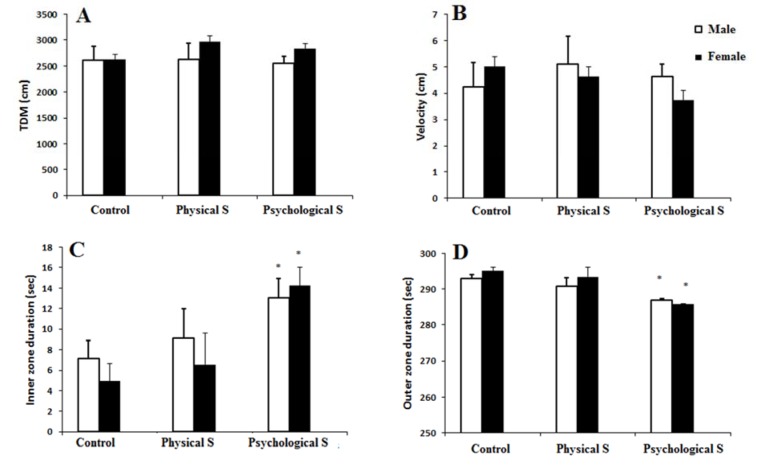
A and B) total distance moved (TDM) and velocity were not statistically different among groups of study. C and D) A decreased anxiety-like behavior was observed in the psychological prenatal stress group in the open field paradigm. * p < 0.05 as compared to the control group

In the open field test, no significant difference in total distance moved and velocity was observed which implies that there is no impaired motor function observed in the stressed animals in comparison to the control rats (Figure 2A and B(Fig. 2)). 

Male and female offspring of the psychological stress group had a longer duration of staying in the center in comparison to the control group which is an indirect indicator of anxiolytic-like effects (p < 0.05; Figure 2C and D(Fig. 2)).

### Elevated plus maze

**Figure 3:**
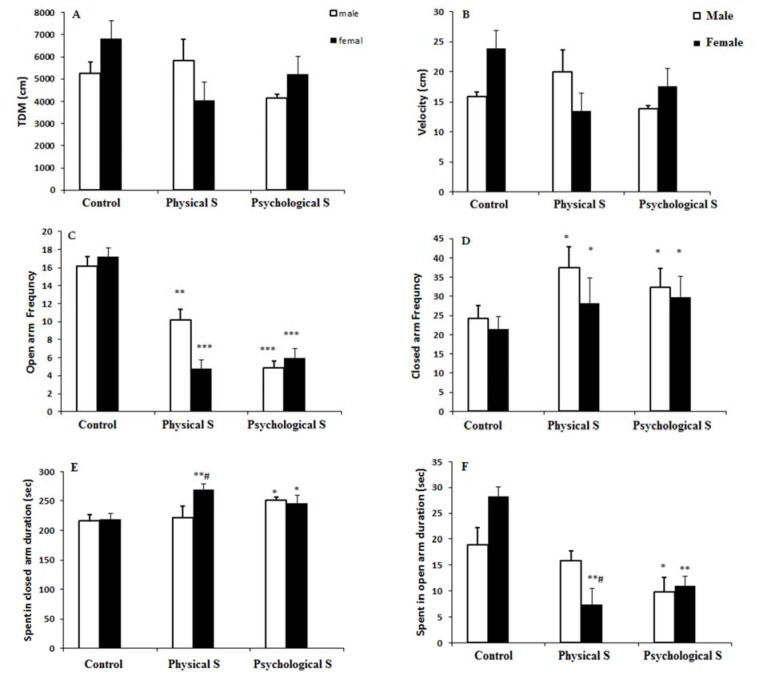
A and B) Total distance moved and velocity were not different among groups of study. C) Number of entrance into the open arm was significantly diminished in offspring of the physical and psychological stress in comparison to the control animals. Furthermore, time spent in open arms in a five minute duration was significantly reduced in physical stress female offspring and psychological stress male and female offspring in comparison to the control group. D) Number of entrance into the closed arm was significantly increased in offspring of the physical and psychological stress groups in both male and female rats. E) Time spent in closed arm was significantly increased in male offspring of the physical stress group and both male and female offspring of psychological stress group as compared to the control group. F) Time spent in open arm was significantly decreased in female offspring of physical stress group and both male and male of psychological stress group. * p < 0.05, ** p < 0.01, *** p < 0.001 as compared to the control group. # p < 0.05 as compared to the female offspring of the physical stress group animals.

Total distance moved (TDM) and velocity did not differ among different groups of stress in both genders which implies a non-altered motor function in stressed groups in comparison to the on-stressed rats (p > 0.05; Figure 3A and B(Fig. 3)). 

Number of entrance into the open arm was significantly reduced in both male and female offspring of physical and psychological stress groups, implying a higher level of anxiety in these two subgroups (p < 0.01). There was no significant difference among physical and psychological stress offspring (p > 0.05; Figure 3C(Fig. 3)).

The number of entrance into the closed arms was increased in offspring of both physical and psychological stress groups in comparison to control rats (p < 0.05). No significant differences were observed among two groups of physical or psychological stress regarding the number of entrance into the open arms (p > 0.05; Figure 3D(Fig. 3)).

Time spent in open arm was significantly reduced in female offspring of physical stress group and both male and male offspring of psychological stress group (P < 0.05). Male offspring of physical stress did not have a significant difference in time spent in open arm in comparison to control subjects and there was a significant difference in time spent in open arm between male and female offspring of physical stress group (p < 0.05; Figure 3E(Fig. 3)).

Time spent in close arms was increased in female offspring of physical stress group and male and female offspring of psychological group (p < 0.05, ANOVA). There was also a significant difference between male and female offspring of physical stress group, so that the male offspring did not differ regarding the time spent in open arms in comparison to control group (Figure 3F(Fig. 3)). 

### Motor function, balance and muscle strength 

**Figure 4:**
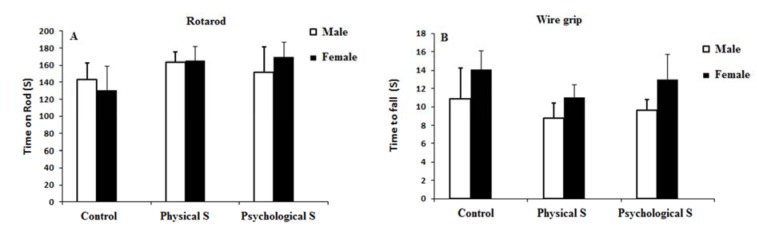
A and B) Time on rod and time to fall in rotarod and wire grip tests were not different among groups of study which indicates a non-impaired balance and muscle strength in the animals.

There were no significant difference observed among groups of study regarding the time on rod and time to fall in rotarod and wire grip tests respectively (p > 0.05; Figure 4A and 4B(Fig. 4)).

### Morphine conditioned place preference

**Figure 5:**
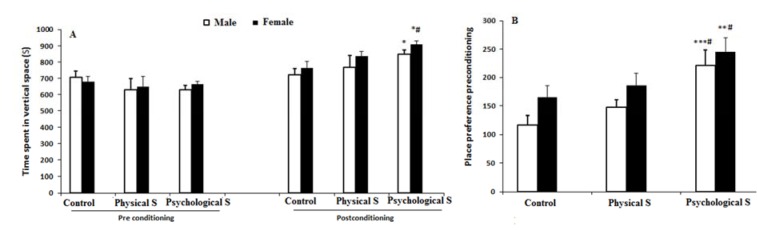
Conditioned place preference score (CPP) of the psychological stress group offspring was significantly increased in comparison to the control and physical stress groups which indicates an increased preference for morphine in the psychological stress offspring. * p < 0.05, ** p < 0.01, *** p < 0.001 as compared to the control group, # p < 0.05 as compared to the physical stress female offspring. Two way ANOVA followed by Tukey's post-hoc test

The CPP score was compared among groups of study. Offspring of the physical stress group did not have a significant difference with control subjects regarding CPP score, while offspring of psychological stress group had an increased CPP score in comparison to both control and physical stress offspring groups (p < 0.05, Two-way ANOVA followed by Tukey's post-hoc analysis) (Figure 5A and B(Fig. 5)).

## Discussion

Results of the current study revealed that both physical and psychological prenatal stress increased anxiety-like behavior in animals in the EPM paradigm. Prenatal stress did not alter the motor function, balance or muscle strength in offspring during their adolescence and rats exposed to psychological stress during fetal development had an increased tendency to morphine as demonstrated in conditioned place preference paradigm. 

Results of the current study highlight the importance of the type of stressor experienced during fetal period on development of addictive behaviors in adolescence. Previous studies have demonstrated the effect of stress during pregnancy on cognitive function of offspring. Vallee et al. (1997[43]) have shown that exposure of the pregnant animal to stress induces anxiety-like behaviors in offspring (Vallée et al., 1997[43]), which is confirmed in the current study. Offspring of both physical and psychological stress groups had an increased anxiety-like behavior as revealed by EPM paradigm. Human and animal studies have demonstrated the devastating effect of stress hormones on fetal development which leads to several impairments in offspring (Welberg and Seckl, 2001[44]; Kofman, 2002[15]; Schneider et al., 2002[35]; Laplante et al., 2004[18], 2008[19]). There are several mechanisms proposed for such observations, of the most importance is the role of stress hormones on fetal development. An adequate level of stress hormones is essential for proper development of fetus nervous system, but excess hormones might lead to dysfunction in structure and function of the neural cells under development (Lemaire et al., 2000[20]; Maccari et al., 2003[22]). In the current study, a specific point in development was set for evaluation of addictive behaviors. Adolescence was chosen due to the fact that many addictive behaviors initiate at this age and thus, prenatal stress might alter the animals' preference for drugs at this time points. Besides, anxiety-like behaviors have also been shown to alter during development (Lynn and Brown, 2010[21]), thus anxiety-like behaviors were also evaluated in the adolescence of the animals as well. 

Animals in both physical and psychological stress group did not differ in their motor function, balance and muscle strength which demonstrates that observations made in EPM and OFT paradigms are not related to motor dysfunction in the animals. Consistent with the current study, we have already demonstrated that locomotion of offspring exposed prenatally to physical or prenatal stress is not significantly altered. Therefore, it seems reasonable to conclude that findings in anxiety assays are related to anxiety-like behavior itself and not due to the changes in motor function (Nazeri et al., 2015[26]). There was a difference in two anxiety assays. While both physical and psychological stress offspring had an increased anxiety-like behavior in the EPM paradigm, findings of the open field test were different. Many factors could contribute to this difference, including the timing of the tests and environmental changes during these assays. Open field is a valid model of locomotion and anxiety-like behavior (Prut and Belzung, 2003[29]), but it has some limitations as well. Thus, our emphasis was to check for locomotion and explorative behavior changes in the studied groups and EPM was recruited to assay anxiety-like behaviors.

The most important finding of the current study was that psychological stress significantly alters preference in both male and female offspring, which is the novel finding of the current study. Interestingly, offspring of the physical stress group did not have a significant increase in tendency towards morphine. Conditioned place preference is a well-established method of evaluating the tendency to a specific agent, morphine in the current study. Previous studies have demonstrated an increased preference or self-administration of different drugs in CPP paradigm in animals exposed to prenatal stress. Yang et al. (2006[45]) evaluated the effect of prenatal exposure to foot shock stress on addictive and depressive-like behaviors of offspring (Yang et al., 2006[45]). They demonstrated that unpredictable electric foot shock applied in gestational days 13 to 19 enhances the place preference for morphine which is consistent with our findings regarding the psychological stress offspring. They also demonstrated that enriched environment counteracts the effects of prenatal stress. Though we did not evaluate a treatment modality in the current study, but the finding that different stressors administered during pregnancy have different effect on addictive behavior is noticeable since it might provide evidence for the role of stress type on addictive behavior in offspring (Yang et al., 2006[45]). 

Regarding the mechanism of prenatal stress effect on addictive behavior of offspring, Sanchez et al. (1996[34]) have demonstrated that µ-opioid receptor density is decreased in the brain of animals exposed prenatal restraint stress. This finding might provide some explanations for the current study, so that a reduced number of opioid receptors might lead to the enhanced need for opioids and therefore would be responsible for drug craving and tolerance (Sanchez et al., 1996[34]). In another study by Kippin et al. (2008[13]), they evaluated the effect of prenatal restraint stress on responsiveness to cocaine. Prenatal stress led to an increase in tendency towards cocaine and a lowered level of extinction (Kippin et al., 2008[13]). One limitation to the current study is that we did not evaluate the effect of prenatal stress on extinction in morphine conditioned place preference which should be evaluated in further studies. 

There are human and animal studies concerning the role of gender in stress-induced neural changes and addiction (Thomas et al., 2009[41]; Chaplin et al., 2010[5]; Swendsen and Le Moal; 2011[40]). In the current study, both male and female offspring of psychological stress rats had an increased preference for morphine. This finding might implicate that both genders are vulnerable to prenatal psychological stress and it might have a great impact on addictive behavior of the animals. Thomas et al. (2009[41]) have shown that prenatal restraint stress affects both male and female offspring addictive behavior to cocaine, but males are more prone to the reinforcing effects of cocaine, while females are more susceptible to the psychomotor sensitizing effects of the drug. According to the current study and Thomas et al. (2009[41]) study, it seems that the stressor type applied during pregnancy has an important role in addictive behavior of the offspring (Thomas et al., 2009[41]).

## Conclusion

Results of the current study demonstrated that exposure to physical and psychological stress during fetal development leads to an increased anxiety-like behavior in the adolescent as revealed by EPM paradigm and psychological prenatal stress makes offspring of both sexes vulnerable to morphine exposure. Further studies to evaluate the role of sex hormones and different durations of stress on addictive behavior of offspring might help to better clarify the exact mechanisms and to help provide preventive measurements.

## Conflict of interest

The authors declare no conflict of interest. 

## Acknowledgement

Funding for this study was provided by Neuroscience Research Center, Neuropharmacology Institute, Kerman University of Medical Sciences, Kerman, Iran. The results described in this paper were part of MD student thesis.
